# A cross-sectional survey of the safety attitudes questionnaire subscale stress recognition and the effect of non-technical skills training in operating theatre staff

**DOI:** 10.1515/iss-2024-0025

**Published:** 2024-11-20

**Authors:** Sebastian Leuschner, Philipp Schenk, Carolin Gräbsch, Frank Siemers

**Affiliations:** Department of Plastic and Hand Surgery, 39781 Burns Unit, BG Klinikum Bergmannstrost, Halle, Germany; Department of Science, Research and Education, 39781 BG Klinikum Bergmannstrost, Halle, Germany; Department of Plastic and Hand Surgery, Burns Unit, BG Klinikum Bergmannstrost, Halle, Germany; Martin Luther University Halle-Wittenberg, Halle, Germany

**Keywords:** non-technical skills training, safety attitudes questionnaire, stress recognition, leadership

## Abstract

**Objectives:**

Non-technical skills (NTS) training of surgical staff is a quality improvement measure to improve patient safety. One factor that can compromise patient safety is impaired staff performance due to stressors such as tiredness. Awareness of the impact of such stressors is measured by the stress recognition (SR) domain of the Safety Attitudes Questionnaire (SAQ). The aim of this study was to assess whether NTS training improves SR scores and whether there are any groups with divergent SR scores.

**Methods:**

A cross-sectional survey of all operating theatre staff in a German major trauma centre was undertaken using the stress recognition (SR) subscale of the SAQ. A multivariable linear regression was performed to assess which factors are associated with SR scores. Data are presented as median (interquartile range).

**Results:**

From 226 invited staff members, 89 responses (39 %) were received. Twenty-eight respondents (31 %) had attended NTS training. The overall SR score was 4.3 (3.5–4.5). There was no effect of NTS training, age, gender, profession, or specialty on SR scores. Physicians in leadership positions had lower SR scores (3.4 (3.06–4.0)) than physicians without leadership positions (4.5 (4.3–5.0), p<0.001).

**Conclusions:**

Attendance at NTS training courses did not improve SR scores in the clinical staff of operating theatres of a German major trauma centre. Interestingly, physicians in leadership positions had lower SR scores than other physicians. Further studies using the SAQ should discriminate between physicians in leadership positions and other physicians when reporting SR scores.

## Introduction

Non-technical skills (NTS) training (also known as crew resource management training) has been adapted from aviation for surgical staff to improve patient safety in the operating theatre [1]. Non-technical skills encompass situational awareness, decision making, team work, communication and leadership [2].

Impaired performance due to tiredness or excessive stress (e.g., due to workload or a hostile working environment) are factors that can jeopardise patient safety. Awareness of these factors, that affect one’s own but also others’ performance, i.e. stress recognition, is a core component of NTS training [2]. For these reasons attendance at NTS training courses are encouraged in our hospital.

The Safety Attitudes Questionnaire (SAQ) is a validated tool also adopted from aviation [3]. It evaluates safety climate by quantifying staff perceptions of and attitudes towards health and safety attitude. The SAQ has six domains: teamwork climate, safety climate, job satisfaction, stress recognition, perceptions of management and working conditions. The stress recognition (SR) domain measures the participant’s awareness of factors that (negatively) affect their own performance. One would therefore expect that SR scores improve after NTS training. However, in a large retrospective study of NTS training the SR subscale improved in only one of 63 surveyed units [4]. However, the intervention in this study (medical team training) focussed on improving patient safety culture. It is unclear how much, if any, emphasis was given to stress recognition and management, which might explain the lack of effect seen.

The other five domains of the SAQ measure the participants’ perceptions and attitudes towards the safety climate of an entire unit, e.g. the quality of teamwork. Previous studies have shown that these five domains correlate with each other, with an r-value between 0.6 and 0.8^5^. The SR domain does not correlate as well (r −0.1 to −0.2) with the other five domains [5], 6] because the questions of the SR domain are about awareness of factors that affect the safe performance of the individual, whereas the questions of the other domains are concerned with the safety climate in the unit [7].

The primary objective of our study was to evaluate whether clinical staff in our operating theatres who have received NTS training, have higher stress recognition scores than those who had no training. The secondary objective was to identify subgroups who may have particularly high or low SR scores.

## Methods

### Survey

A cross-sectional survey of all clinical staff working in our operating theatres was conducted between 28.11.2019 and 31.01.2020 using the previously validated German version of the SAQ [8]. For our study, only the stress recognition (SR) domain was used. Briefly, each question is scored on a 5-point-Likert scale ranging from 1 (very low awareness of the impact of stressors) to 5 (very high awareness). The four items are listed in Table 1. The SR score is obtained by calculating the mean score of the four items in the domain.

**Table 1:** Items of the stress recognition subscale of the safety attitudes questionnaire.

Stress recognition
When my workload becomes excessive, my performance is impaired.
I am less effective at work when fatigued.
I am more likely to make errors in tense or hostile situations.
Fatigue impairs my performance during emergency situations (e.g. emergency resuscitation, seizure).

Further questions were included about demographics (sex and age), profession (anaesthetic nurse, scrub nurse, anaesthetist, surgeon), specialty (general surgery, neurosurgery, plastic surgery, trauma and orthopaedic surgery, urology), duration of professional experience, for physicians (anaesthetists and surgeons), whether they hold a leadership position, and if NTS training had previously been attended.

To protect the anonymity of respondents, age was recorded in decades. Regarding positions of leadership, physicians were divided in two groups: physicians who regularly supervise other physicians (“Oberarzt” and “Chefarzt” in the Germany medical system, roughly equivalent to consultant and head of department in the UK system) and physicians who do not regularly supervise other physicians.

### Participants

The study was carried out at the BG Hospital Bergmannstrost, a major trauma centre with 580 beds and 11 operating theatres. All clinical staff working in the operating theatres were eligible for inclusion in the study.

Eligible physicians were contacted by email on their professional email accounts and invited to participate in the survey hosted on LamaPoll (Lamano, Berlin, Germany). Reminder emails were sent after two and six weeks.

Nursing staff do not have email accounts at our institution. Therefore, the surveys were printed and distributed at the weekly departmental meetings of the scrub and anaesthetic nursing staff. After two and six weeks, meetings were attended again to encourage staff to complete the surveys. After 13 weeks the online and offline survey was closed, and the paper versions of the surveys were digitalised.

### Statistical analysis

Results of the SAQ are non-parametric and were therefore reported as median (interquartile range, IQR). Univariable and, where appropriate, multivariable linear regression analyses was performed with SR score as the dependent variable and participation at NTS training, age, sex, professional group, specialty, and leadership position as independent variables. Since the variables specialty and leadership position only apply to physicians, a separate linear regression was performed for physicians.

To test for differences in NTS training attendance across demographic subgroups Fisher’s exact test was used. To detect a possible wash-out effect of NTS training a linear regression of SR scores using time since last NTS training as the independent variable was performed.

For comparisons of two groups a Mann–Whitney U test was used.

The significance level was set at 0.05. R [9] with R Studio (version 1.2.5033) was used for statistical analyses.

### Ethical considerations

Participants were informed of the purpose of the study and asked to give their consent for their participation prior to completing the survey. All responses were collected anonymously.

The study was assessed and approved by hospital management, the Data Protection Officer, the Department of Science, Research and Education, and employee representation of the BG Hospital Bergmannstrost. No additional ethical approval was required.

## Results

### Demographics

A total of 226 staff members were invited to participate. From these 89 responses were received, resulting in a response rate of 39 %. The response rate among the professional groups was 47 % (22/47) for anaesthetists, 43 % (21/49) for scrub nurses, 36 % (13/36) for anaesthetic nursing staff and 34 % (32/94) for surgeons. Among physicians, the response rate of physicians in leadership roles was 47 % (18/38) and the response rate of all other physicians 34 % (31/92).

Forty-five (51 %) of the respondents were female. A summary of participants’ demographics across the two groups of those with and without NTS training is shown in Table 2. There were no significant differences in demographics between the two groups.

**Table 2:** Respondent demographics, stratified by attendance at an NTS training course.

Attendance at NTS training course		No (n, %)	Yes (n, %)	p-Value
Age (years)	21 to 30	10 (16 %)	1 (4 %)	0.14
	31 to 40	17 (28 %)	8 (29 %)	
	41 to 50	16 (26 %)	14 (50 %)	
	51 to 60	16 (26 %)	5 (18 %)	
	>60	2 (3 %)	0 (0 %)	
Gender	Female	33 (54 %)	12 (43 %)	0.70
	Male	26 (43 %)	16 (57 %)	
	Other	1 (2 %)	0 (0 %)	
	Not specified	1 (2 %)	0 (0 %)	
Profession	Anaesthetic nurse	9 (15 %)	4 (14 %)	0.80
	Scrub nurse	16 (26 %)	5 (18 %)	
	Anaesthetist	13 (21 %)	9 (32 %)	
	Surgeon	22 (36 %)	10 (36 %)	
	Not specified	1 (2 %)	0 (0 %)	
Professional experience (years)	<1	3 (55 %)	0 (0 %)	0.37
	1 to 2	1 (25 %)	0 (0 %)	
	3 to 5	7 (12 %)	2 (7 %)	
	6 to 10	12 (20 %)	3 (11 %)	
	11 to 20	12 (20 %)	11 (39 %)	
	>20	26 (43 %)	12 (43 %)	
Leadership role	Physicians in leadership role	9 (26 %)	9 (47 %)	0.32
	Physicians without leadership role	22 (63 %)	9 (47 %)	
	Not specified	4 (11 %)	1 (5 %)	
Surgical specialty	General surgery	3 (14 %)	4 (40 %)	0.07
	Neurosurgery	2 (9 %)	2 (20 %)	
	Plastic surgery	8 (36 %)	1 (10 %)	
	Trauma and orthopaedic surgery	9 (41 %)	2 (20 %)	
	Urology	0 (0 %)	1 (10 %)	

The p-value refers to the result of Fisher’s exact test to detect differences between groups.

Nearly one third of the 89 respondents (n=28, 31 %) had attended NTS training. Eighteen (64 %) had attended an NTS course delivered by Lufthansa Aviation Training GmbH, Munich, Germany. Five respondents had attended other courses and five did not specify which course they had attended.

### Stress recognition and NTS training

Across the entire cohort the SR score was 4.3 (3.5–4.5). The SR score of respondents who had received NTS training was 4.3 (3.5–4.5) and the score of those who had not was 4.0 (3.3–4.5). Attendance at an NTS course but also age, gender and profession had no effect on SR scores (Table 3).

**Table 3:** Linear regression of stress recognition (whole dataset).

Independent variable	Level	n	Median	IQR	Coefficient (univariable)
Attendance at NTS training course	No	59	4.0	3.3 to 4.5	–
	Yes	28	4.3	3.5 to 4.5	0.12 (p=0.53)
Age (decade)	3 to 7	87	4.3	3.5 to 4.5	−0.13 (p=0.15)
Gender	Female	45	4.3	3.5 to 4.5	–
	Male	42	4.0	3.3 to 4.5	0.04 (p=0.82)
Profession	Anaesthetic nurse	13	3.5	3.0 to 4.0	–
	Scrub nurse	21	4.3	3.3 to 4.5	0.46 (p=0.12)
	Anaesthetist	22	4.3	4.0 to 4.5	0.53 (p=0.07)
	Surgeon	31	4.1	3.5 to 4.6	0.46 (p=0.10)

Age is given as decade of life (e.g., 3rd decade: 21 to 30).

A sensitivity analysis excluding those respondents who had not attended their NTS course at Lufthansa Aviation Training also showed no differences in SR scores between those who had attended training (4.0 (3.5–4.5)) and those who had not (4.0 (3.3–4.5), univariable coefficient=0.09, p=0.71).

It was explored whether the lack of effect of NTS training could be due to a wash-out effect over time. However, no relationship between SR score and time since last NTS training was observed in linear regression (p=0.78).

### Subgroup analysis of physicians

Among physicians, attendance at NTS training, age, gender, and specialty were not associated with SR scores (Table 4). However, SR scores were almost one point lower in physicians in leaderships roles (3.8 (3.1–4.0)) compared to other physicians (4.5 (4.3–5.0), p<0.001, Table 4). This variable explained 28 % of the variation of SR. Multivariable regression was performed to show that this effect was not explained by participant age. The SR scores of the physicians in leadership roles who had and had not undergone NTS training were 4.0 (3.3–4.3) and 3.8 (3.0–4.0), respectively. In this group there was also no statistically significant effect of NTS training (W=29, p=0.33).

**Table 4:** Uni- and multivariable linear regression of stress recognition (physicians only, n=53).

Independent variable	Level	n	Median	IQR	Coefficient (univariable)	Coefficient (multivariable)
Attendance at NTS training course	No	34	4.0	3.5 to 4.6	–	–
	Yes	19	4.3	4.0 to 4.5	0.11 (p=0.56)	0.44 (p=0.08)
Age, decade	3 to 7	53	4.3	3.6 to 4.5	−0.11 (p=0.20)	0.06 (p=0.69)
Gender	Female	18	4.3	3.6 to 4.5	–	–
	Male	35	4.0	3.6 to 4.6	0.06 (p=0.74)	0.31 (p=0.25)
Leadership role	Physician in leadership role	18	3.8	3.1 to 4.0	–	–
	Physician without leadership role	31	4.5	4.3 to 5.0	0.98 (p<0.001)	1.40 (p<0.001)
	Not specified	5	4.0	3.3 to 4.0	0.11 (p=0.77)	0.15 (p=0.75)
Specialty	General surgery	7	3.4	2.9 to 4.4	–	–
	Neurosurgery	4	4.1	3.8 to 4.3	0.38 (p=0.53)	0.50 (p=0.33)
	Plastic surgery	9	4.0	3.5 to 4.3	0.42 (p=0.40)	0.16 (p=0.72)
	Trauma and orthopedic surgery	11	4.5	4.1 to 4.9	0.67 (p=0.16)	0.63 (p=0.15)
	Anaesthesia	22	4.3	4.0 to 4.5	0.51 (p=0.24)	−0.03 (p=0.93)

Age is given as decade of life (e.g., 3rd decade: 21 to 30). Model fit: R^2^=0.41, Adjusted R^2^=0.28.

## Discussion

### Statement of principal findings

This cross-sectional survey evaluated the effect of non-technical skills (NTS) training on the stress recognition (SR) subscale of the Safety Attitudes Questionnaire (SAQ) in clinical staff working in the operating theatres of a major trauma centre in Germany. No differences in SR score were found between staff members who had attended NTS training and those who had not. Among physicians SR scores were about one point on a five point Likert scale lower in those who held leadership positions. No other predictors of SR score could be identified.

### Strengths and limitations

An important strength of the study is that it evaluates the SR subscale of the SAQ separately, because it is the only subscale which measures respondents’ self-awareness of factors which may impair performance, whereas the other subscales measure respondents’ evaluation of the safety culture of the entire unit. The former would be expected to change with NTS training of individuals, whereas the latter would not. Another strength of the current study is that it is the first one to show that physicians’ hierarchical position and stress recognition seem to be correlated.

This is a retrospective, monocentric study limiting the generalisability of the results. Further studies in different settings and with larger numbers are needed to verify the results.

Furthermore, the response rate of 39 % was relatively low. However, a low response rate itself is not a predictor of non-response bias [10]. The distribution of response rates across professional groups and between physicians in leadership roles and those without was relatively even.

### Interpretation within the context of the wider literature

In the current study, no evidence for an effect of NTS training on SR scores was found. From a conceptual point of view, this result is unexpected: NTS training aims to improve patient safety by increasing awareness of common causes of errors and strategies to minimise their occurrence [2]. As such, we would expect that NTS training hones the participants’ awareness of the deleterious effect of stress and tiredness on performance. The SR domain of the SAQ measures exactly this (self-)awareness [11]. Therefore, we would expect SR scores to increase after NTS training.

This raises the question to what extent teaching on factors that adversely affect performance is part of the NTS training courses our respondents attended. In their systematic review of NTS training interventions Gross et al. found that the contents and delivery of NTS interventions are often poorly described [12]. They also found significant heterogeneity in the skills and topics covered in different NTS training courses and concluded that NTS training can better be described as an umbrella term rather than a specific intervention.

Since the NTS courses attended by our clinical staff were provided by external organisations, limited information on the content was available to us. However, one of the providers (Lufthansa Aviation Training, Munich, Germany), a subsidiary of a major German airline, performs NTS training for their flight crews. They developed their NTS training course for healthcare providers together with the German Society for Trauma and Orthopaedic Surgery and other medical experts [13]. It can therefore be presumed that they have the expertise necessary to run NTS training courses for healthcare staff. According to the course contents published online, these NTS courses address the following topics: “stress and safety” [14], “dealing with fatigue”, “workload management” and “dealing with stress” [15].

Those staff members who attended NTS courses reported attendance at a variety of courses. The quality of these courses is likely to be heterogeneous. However, the majority of our staff had attended a course by Lufthansa Aviation Training. A sensitivity analysis excluding those who had attended other courses also showed no effect of NTS training on SR score.

It is also possible that any effect of training on SR scores may be subject to wash-out over time. If such a time-dependent effect was observed, it could be considered evidence for a training effect of the courses. However, no relationship between time since training and SR scores could be found.

Another possible reason for the lack of effect seen in this study are the already generally very high SR scores across the whole study population. In some of the largest series of the SAQ reported to date SR scores were between 2.78 [16] and 3.79 [4]. Sexton et al. described a mean SR score of 3.19 in 10,842 healthcare professionals when they validated the SAQ in three countries in 2006 [11]. Cui and colleagues translated the SAQ to Chinese and subsequently validated the questionnaire in a sample of 1663 in 2014 [16]. They reported a mean SR score of 2.79. These scores are considerably lower than those reported in our study. It is therefore possible that NTS training had no further effect on our study cohort due to the already high SR scores, even though it might have done if SR scores had been lower.

In a large multicentre study, Watts and colleagues performed the SAQ before and 8 months after NTS training in the operating theatres of 63 different hospitals [4]. Across all hospitals mean SR scores improved from 3.73 to 3.79. However, when examining the sites individually the authors found a statistically significant improvement of SR scores in only one of those sites. In a multidisciplinary obstetric setting, Haller et al. (2008) found NTS training to improve SR after 4 and 8 months in a before and after cross-sectional design [17]. The NTS course used in this study was specifically designed for the obstetric unit in which its effects were evaluated.

The secondary objective of the study was to identify groups with particularly high or low SR scores. There was no evidence that age, gender, profession, or specialty affected SR scores. An interesting finding of the current study was that physicians in leadership roles (“Chefärzte” and “Oberärzte” in the German medical system, roughly equivalent to heads of department and consultants in the UK) had SR scores that were one point lower than those of other physicians on the five point SR scale. Since physicians who hold leadership positions tend to be older than other physicians, a possible explanation might be that the effect is mediated by age. However, the effect of leadership position on SR score was unaffected when controlling for age.

Another possible explanation for the differences in SR scores between the groups might be a generational effect. However, the fact the relationship between SR scores and leadership position were robust when controlling for age, make a generational effect less likely. Due to the small sample size this may be a question to be addressed in future studies.

A subgroup analysis of physicians in leadership roles also found no effect of NTS training on SR scores although this finding must be interpreted cautiously due to the small sample size (n=18) of this group. Nevertheless, it suggests that the generally high SR scores in this study may not be the sole explanation for the lack of improvement after NTS training.

### Implications for policy, practice and research

While the finding that physicians in leadership positions have lower SR scores is preliminary and requires replication in a larger and ideally multicentric study, its implications are of interest. Physicians holding these leadership roles have greater influence over management decisions, such as whether or not a patient requires surgery out of hours. If these physicians have a lower awareness that tiredness, for instance, impairs performance, they may be less likely to take account of these factors when scheduling urgent surgeries.

Our study also raises the question what the reason for this difference in SR scores may be. Further studies might explore whether the reduction in SR scores is acquired when physicians take up leadership roles or whether physicians with lower SR scores are more likely to be appointed to leadership positions. Another interesting question is whether physicians in leadership roles also believe their subordinates to be less vulnerable to fatigue and stress.

Further work may focus on whether information on factors impairing performance is part of the content of NTS training courses. A better understanding of any potential effect of NTS training on SR scores can be expected from a prospective study. Multicentric studies would also be able to evaluate whether NTS training has other beneficial effects on safety climate by evaluating the other domains of the SAQ. Future studies using the SAQ to evaluate safety climate should examine SR scores in physicians with and without leadership positions.

Further research might also aim to more clearly define the aims and contents on NTS courses. Courses that are designed for specific professional groups may be more effective than courses generally aimed at healthcare professionals.

### Conclusions

In conclusion, this study failed to show an effect of NTS training on SR scores. While gender, age, profession, and specialty did not affect SR scores, SR scores of physicians in leadership roles were lower than those of other physicians which, if replicated, has important implications for patient safety.
